# EMG-Phänomene myogener Übererregbarkeit

**DOI:** 10.1007/s00115-023-01597-y

**Published:** 2024-01-09

**Authors:** Andreas Posa, Malte Kornhuber

**Affiliations:** 1 Universitätsklinik für Neurologie, Martin-Luther-Universität Halle-Wittenberg, Halle (Saale), Deutschland; 2 Klinik für Neurologie, Helios Klinik Sangerhausen, Sangerhausen, Deutschland

**Keywords:** Elektrophysiologie, Nadelelektromyographie, Pathologische Spontanaktivität, Neuromuskuläre Erkrankungen, Myogene Übererregbarkeit, Electrophysiology, Needle Electromyography, Pathologic Spontaneous Activity, Neuromuscular Diseases, Myogenic Hyperexcitability

## Abstract

Art, Verteilungsmuster und der zeitliche Verlauf muskulärer Spontanaktivität sind für die Diagnostik neuromuskulärer Krankheiten im klinischen Alltag bedeutsam. Bei neurogenen Läsionen mit motorisch axonaler Beteiligung ist pathologische Spontanaktivität (PSA) meist 2 bis 4 Wochen nach Läsionsbeginn mittels Nadelelektromyographie sicher fassbar. Das Verteilungsmuster korreliert dabei mit dem Läsionsort. Schwerpunkt der vorliegenden Arbeit liegt in der Darstellung der unterschiedlichen PSA-Verteilungsmuster bei myogenen Erkrankungen.

## Abstract (en)

The type, distribution pattern and time course of spontaneous muscular activity are important for the diagnostics of neuromuscular diseases in the clinical practice. In neurogenic lesions with motor axonal involvement, pathologic spontaneous activity (PSA) is usually reliably detectable by needle electromyography (EMG) 2–4 weeks after occurrence of the lesion. The distribution pattern correlates with the lesion location. The focus of the present work is the description of the different forms of PSA in myogenic diseases.

## Hintergrund

Zur Diagnostik neuromuskulärer Erkrankungen (NME) spielen elektrophysiologische Untersuchungen, insbesondere die Nadelelektromyographie (EMG) eine wesentliche Rolle. Die EMG bietet dabei die Möglichkeit zur Differenzierung zwischen myogen und neurogen bedingten Erkrankungen. Primär myogene Spontanaktivität (SA) wird unabhängig vom auslösenden Prozess (neurogen/myogen) in der Regel von jeweils einer einzelnen Muskelfaser generiert. SA hebt sich im entspannten Muskel klar und deutlich von der Grundlinie ab und ist daher sehr sensitiv, um eine mit SA einhergehende NME zu detektieren. Hierbei ist die myogene SA besonders sensitiv, um eine NME (z. B. Muskeldystrophie, MD) zu identifizieren. Ferner können aus Art und Verteilungsmuster der SA Schlüsse auf die Pathogenese einer Erkrankung gezogen werden. Bei Gesunden zeigt sich in Ruhe in der EMG physiologisch keine elektrische Aktivität, es sei denn der Muskel wird durch die Nadelelektrode (NE) mechanisch gereizt [1].

Bei Neuropathien, die mit motorisch-axonaler Degeneration einhergehen (motorisch axonale Degeneration), folgt die myogene SA den gut bekannten Verteilungsmustern der nervalen Innervation, was genutzt werden kann, um den Ort der Läsion genauer zu lokalisieren. Der Läsionsort kann bei Mononeuropathien einseitig und sehr umschrieben vorliegen, bei Polyneuropathien (PNP) z. B. einem distal-symmetrischen Muster folgen (z. B. subakute Polyneuropathien, PNP) oder aber etwa auch bei Vorderhornprozessen ubiquitär verteilt auftreten. Bei Myopathien zeigt die SA in der Regel ein bilaterales Befallsmuster in der jeweils stärker betroffenen Muskulatur. Zudem gibt es spezielle Phänomene, wie etwa perkussionsinduzierte Wulst- bzw. Wellenbildungen, wie etwa beim sog. Rippling, ohne oder auch mit SA in der EMG [2]. Auch das Fehlen von SA kann diagnostisch wichtig sein, wie z. B. bei der Steroidmyopathie, bei der es typischerweise nicht zu pathologischer Spontanaktivität kommt (z. B. steroidinduzierte Myopathie [3]).

Folgende Formen myogener SA werden nachfolgend betrachtet: Fibrillationen (Fi), positive scharfe Wellen (PSW), myotone Entladungsserien (ME), hochfrequente einfach-repetitive Entladungsserien (HERE), komplex-repetitive Entladungen (KRE), pathologisch verlängerte Einstichaktivität, perkussionsinduzierte Phänomene. Ein Schwerpunkt der vorliegenden Übersichtsarbeit liegt dabei bei dem Verteilungsmuster pathologischer (myogener) Spontanaktivität (PSA) bei neurogenen bzw. myogenen Krankheitsprozessen.

## Reizinduzierte myogene Phänomene

### Einstichaktivität

**Abb. 1:**
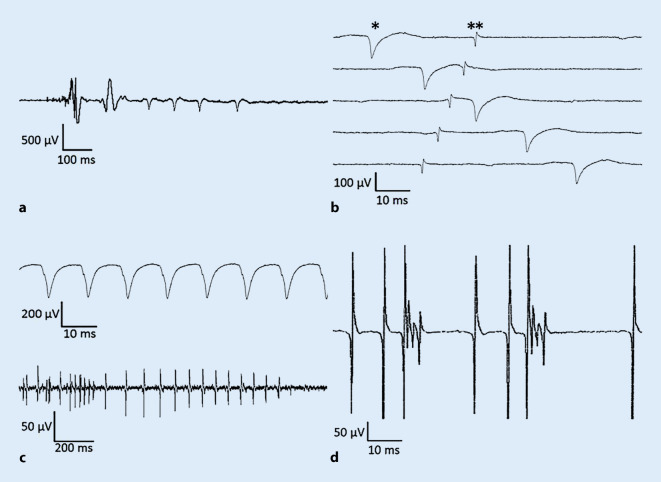
Formen myogener Spontanaktivität. **a** Verlängerte Einstichaktivität, **b** positiv scharfe Wellen (*Stern*) und Fibrillationen (*Doppelstern*), **c** Serienentladungen: *oben* hochfrequente einfach-repetitive Entladungen, *unten* myotone Entladungen, **d** komplexe repetitive Entladungen. *ms* Millisekunden, *µV* Mikrovolt

Beim Bewegen der in den Muskel eingestochenen NE reizt diese mechanisch die Muskelfasermembran, was zu einer Depolarisation und somit zu einem Aktionspotenzial (AP) an der Membran führt [1]. Diese elektrischen Signale lassen sich in der EMG als sog. Einstichaktivität (EA) nachweisen [1]. Bei gesunden Muskeln besteht die EA aus einzelnen AP der mechanisch gereizten Muskelfasern. Sie korreliert zeitlich mit der Dauer der Nadelbewegung und dauert oft um die 50 ms und zumeist weniger als 230 ms ([4]; Abb. 1a, erster Einstich).

**Abb. 2:**
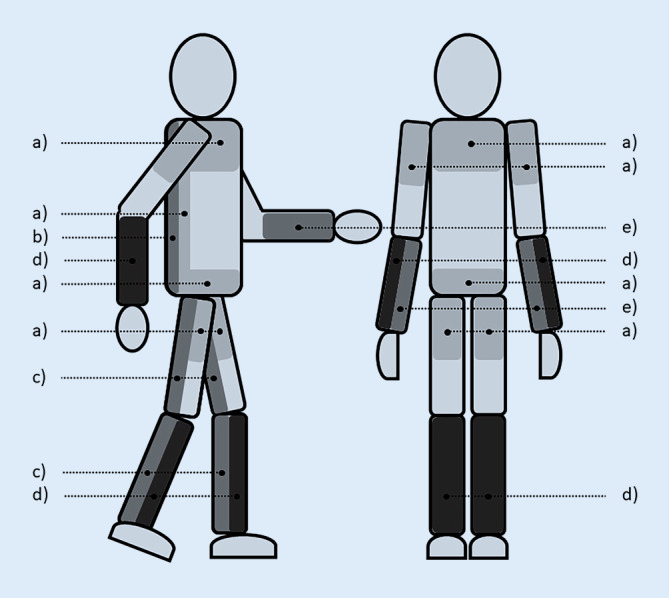
Diagnostisch relevante Schwerpunkte pathologischer Spontanaktivität. *a)* Gliedergürtel-Typ (Polymyositis, Dermatomyositis, Mischkollagenose [Einzelfälle]), *b)* axial-prädominanter Typ (PROMM, myofibrilläre Myopathien [ZASP, Desmin, Myotilin, Filamin C etc.], zentronukleäre Myopathie, Pompe [40], Matrin‑3 [41]); distal-prädominanter Typ: *c)* dorsale Beinmuskulatur: Miyoshi (Dysferlin, Anoctamin 5); *d)* Fingerstrecker, Fußheber: Welander (initial ohne PSA!), Laing; *e)* Fingerbeuger, Einchlusskörpermyositis, ohne pathologische Spontanaktivität (Udd [Tibialis-anterior-Myopathie])

#### Veränderte Einstichaktivität

Eine vermehrte EA liegt vor, bei einem anhaltenden, jedoch zeitlich befristeten Feuern von Spikes nach Beendigung der Nadelbewegung und deutet auf eine erhöhte Reizbarkeit und/oder eine Instabilität der Muskelfasermembran hin. Sie kann sowohl bei neurogenen [5] als auch bei myogenen [6] Störungen auftreten (Abb. 1a, zweiter Einstich). Selten wurde eine verlängerte EA auch in gesunden Muskeln beschrieben, vor allem bei jungen muskulösen Männern und dabei öfter in der Bein- als in der Armmuskulatur [7]. Beim Verlust von Muskelfasern, beim Ersatz des Muskels durch Bindegewebe oder bei einer Untererregbarkeit der Muskelfasermembran kann sich eine verringerte EA zeigen. Dies kann bei schweren neurogenen oder myogenen Störungen auftreten [8]. Bei ischämischen Muskeln kann die EA aber auch ganz fehlen (z. B. Kompartmentsyndrom [9]).

### Perkussionsinduzierte myogene Phänomene

Perkussionsinduzierte Muskelantworten sind bekannte EMG-Phänomene, etwa bei der myotonen Dystrophie Typ 1 [10]. Hier lassen sich auf Perkussion der Muskulatur mit EMG myotone Entladungsserien registrieren. Seltener sind die perkussionsinduzierten Wulst- oder Wellenbildungen („percussion induced muscle mounding“, PIMM; „percussion induced rippling contractions“, PIRC) bei der sog. „rippling muscle disease“ (RMD; [2]). Diese Phänomene bleiben im EMG zumeist stumm. Nur selten gehen sie mit Fibrillationspotenzialen, PSW oder ME einher [11].

## Formen myogener Spontanaktivität

Die vorliegende Übersicht ist zwar der myogenen SA gewidmet. An dieser Stelle lohnt es aber, zunächst auf die Analyse der Potenziale motorischer Einheiten (PME) im EMG zu schauen. Bei myogenen Erkrankungen (z. B. MD) lässt sich der Muskelfaserverlust in den motorischen Einheiten mittels EMG mehr oder minder gut nachweisen. In Abhängigkeit von der Ausprägung der Erkrankung zeigen sich hier PME mit verminderter Amplitude, verkürzter Dauer und teils vermehrten Turns und Phasen. Bei neurogenen Erkrankungen zeigen sich in der EMG als Folge axonaler Aussprossung zunächst aufgesplitterte breite PME mit normaler Amplitude. Mit fortschreitender Reinnervation finden sich im Verlauf dann pathologisch hohe PME-Amplituden als Zeichen des neurogenen Umbaus. Nach einer motorisch axonalen Läsion und auch bei einer Reihe von Myopathien zeigt sich zudem regelmäßig eine PSA.

Während die Abgrenzung leichter muskeldystrophischer Erkrankungen vom normalen Befund anhand von Konfiguration und Rekrutierung der PME schwierig sein kann, ist die PSA-Detektion recht sensitiv. Dabei ist es leicht möglich, das muskuläre PSA-Befallsmuster zu kartieren und dessen zeitlichen Verlauf zu dokumentieren. Verschiedene Formen der SA in der EMG wurden bisher beschrieben. Einige finden sich in gesunden Muskeln (z. B. Endplattenaktivität, Faszikulationen, die nicht myogenen Ursprungs sind [12]), andere finden sich bei pathologisch veränderten Muskeln oder an der reinnervierenden motorischen Einheit [13].

Die SA kann anhand ihrer Wellenform charakterisiert werden (Amplitudenform: biphasisch, triphasisch; Phasenrichtung: negativ, positiv) und tritt ganz überwiegend regelmäßig auf (a) ohne wesentliche Änderung des Interspike-Intervalls (z. B. KRE), (b) mit linearer Änderung im Interspike-Intervall (z. B. Fibrillationen) oder (c) mit charakteristischer Änderung im Interspikeintervall (z. B. ME).

### Fibrillationen, positive scharfe Wellen

Spontane AP einzelner Muskelfasern lassen sich mit der extrazellulär gelegenen NE als Fi bzw. als PSW registrieren. Bei Fi und PSW handelt es sich um dasselbe AP. Durch leichte Nadelbewegungen im Muskel lassen sich Fi in PSW überführen und umgekehrt. Die mehrheitliche Auffassung ist, dass Fi unter der NE hindurchgehen, während PSW an der NE enden. Es wurde allerdings auch die Auffassung vertreten, dass der Unterschied in der Potenzialkonfiguration auf Gewebsfiltereffekte zurückgeht [14].

Fi und PSW sind die häufigste Form der PSA. Sie werden bei einer Vielzahl von NME beobachtet, etwa bei Denervationsprozessen oder bei primärer Schädigung von Muskelfasern [15]. Bei der Entstehung spielt möglicherweise ein vermindertes Ruhemembranpotenzial durch Abnahme der Kalium- oder Chloridpermeabilität der Muskelfasermembran eine wichtige Rolle [16]. Zudem wurden eine verminderte präsynaptische Acetylcholinproduktion und eine Überempfindlichkeit postsynaptischer Acetylcholinrezeptoren nachgewiesen [15]. Fi und PSW zeigen in aller Regel ein sehr regelmäßiges Auftreten, mit Frequenzen von meist 1–10 Hz [14], einer Dauer von 0,5–5 ms und einer Amplitude von 100–300 mV ([17]; Abb. 1b).

### Serienentladungen

#### Hochfrequente einfach-repetitive Entladungsserien

Wenn Fi oder PSW mit Frequenzen > 10 Hz stetig wiederkehren und dabei oft an Amplitude und Frequenz etwas nachlassen und meist abrupt enden, spricht man von HERE [18]. Erkrankungen, die zu Fi oder PSW prädisponieren, können grundsätzlich auch mit HERE einhergehen. HERE werden überwiegend beobachtet bei der proximalen myotonen Myopathie, der Matrin-3-Myopathie und den sog. myofibrillären Myopathien (z. B. Genmutationen bei Desmin, Myotilin, Filamin C, „Z-band alternatively spliced PDZ motif protein“ [ZASP] [19]; Abb. 1c, oben).

#### Myotone Entladungsserien

ME sind Folge einer Funktionsstörung von Elektrolytkanälen (Natrium, Kalium, Chlorid) in der Muskelfasermembran, mit resultierender Instabilität des Ruhemembranpotenzials [20]. Sie werden durch willentliche Muskelkontraktion, Perkussion des Muskels oder durch Bewegung der NE im Muskel hervorgerufen und entwickeln sich aus kumulativen Nachdepolarisationen, die ausreichen, um repetitive AP derselben Muskelfaser in mehr oder weniger rascher Folge zu generieren [20]. Dabei zeigen sich Spikes, die in einem regelmäßigen, zunehmenden oder abnehmenden Muster auftreten [13], mit Frequenzen zwischen 10–100 Hz ([21]; Abb. 1c, unten). ME treten bevorzugt bei der myotonen Muskeldystrophie Typ 1 sowie bei muskulären Ionenkanalerkrankungen auf [22]. Sie finden sich zudem bei der distalen Myopathie Typ Welander [23] und seltener bei der myotonen Dystrophie Typ 2 sowie bei myofibrillären, kongenitalen und medikamentös induzierten Myopathien [13].

### Komplexe repetitive Entladungen

KRE bestehen aus einer Gruppe unterschiedlicher Potenziale, deren Abfolge zyklisch und mit meist hoher Konstanz und gleichbleibender Frequenz wiederkehrt (Abb. 1d). Die Entladungsfrequenz einer KRE ist regelmäßig, liegt zwischen < 1 und 150 Hz, beginnt und endet oft abrupt und zeigt pro KRE-Zyklus zwischen 2 und mehr als 50 Spikes [24].

Die klinische Bedeutung von KRE ist nicht sicher geklärt. Sie finden sich bei chronisch neurogenen (z. B. Radikulopathie, spinale Muskelatrophie, amyotrophische Lateralsklerose [ALS]) sowie bei chronisch myopathischen (z. B. Muskeldystrophien, M. Pompe) Erkrankungen [25]. Auch ist die Art der Entstehung noch nicht sicher geklärt. Eine Hypothese ist, dass eine einzelne spontan entladende Muskelfaser als Schrittmacher dient, ephaptisch von Muskelfaser zu Muskelfaser fortgeleitet wird und so eine kreisende Erregung unterhält [24]. Eine andere Hypothese geht davon aus, dass die KRE durch den Muskelspindelapparat von afferenten Typ-II-Fasern auf efferente γ‑ und β‑Fasern übertragen werden [26]. Posa und Mitarbeiter haben hingegen kürzlich vorgeschlagen, dass eine spontan entladende einzelne Muskelfaser ephaptisch das Axon bzw. einen Axonzweig einer aussprossenden motorischen Einheit erregt [25]. In diesem Fall wären KRE ein Phänomen aussprossender motorischer Einheiten. Eine kreisende Wiedererregung der Schrittmachermuskelfaser wäre dafür nicht zwingend nötig. Diese Neuinterpretation bzw. Erweiterung der KRE-Pathogenese ist gut vereinbar mit dem Auftreten von KRE bei Erkrankungen, bei denen Reinnervation eine wesentliche Rolle spielt. Dazu gehören zurückliegende Nerven- oder Nervenwurzelläsionen ebenso wie z. B. ubiquitäre Vorderhornprozesse wie die ALS.

## Zeitliche Charakteristik myogener Spontanaktivität

Zum zeitlichen Auftreten und Abebben primär myogener PSA ist relativ wenig bekannt. Bekannt ist, dass Fi und PSW bei einer akuten neurogenen Läsion Tage bis maximal 4 Wochen (i. d. R 2 Wochen) nach Läsionsbeginn elektromyographisch fassbar werden, wobei bei Radikulopathien der früheste Zeitpunkt für das Auftreten von PSA mit 7 Tagen nach Läsionsbeginn beschrieben wurde [27]. Über die Persistenz von PSA nach neurogenen Läsionen gibt es ebenfalls nur spärliche Berichte. Nach geburtstraumatischen Armplexusparesen beim Neugeborenen kann in manchen betroffenen Muskeln die PSA binnen eines halben Jahres sistieren [28]. Bei länger anhaltender PSA kann eine Abnahme der Intensität beobachtet werden. Dabei nimmt die Amplitude von mehr als 1 Jahr nach radikulärer Läsion persistierenden Fi meist auf < 100 µV ab [27]. Die zeitliche Charakteristik von zunächst ausgeprägter PSA, beginnend 2 Wochen nach Läsionsbeginn und einem „Tapering“ in den Monaten danach, kann auch für die Einschätzung der Akuität bzw. der Chronizität einer neurogenen Läsion genutzt werden, insbesondere wenn man PSA mit Potenzialparametern des neurogenen Umbaus motorischer Einheiten in Beziehung setzt. Generell wird angenommen, dass PSA für die Dauer der Reinnervation persistiert und nach abgeschlossener Reinnervation sistiert [29]. Dies schließt ein, dass PSA bei ausbleibender Reinnervation über lange Zeiträume persistieren kann.

## Verteilungsmuster von PSA

### Neurogene Prozesse

Bei relativ frischen umschriebenen peripheren motorischen Läsionen mit axonaler Degeneration, die mehr als 2 Wochen andauern, lässt sich der Läsionsort oft präzise mit PSA lokalisieren. Das PSA-Verteilungsmuster folgt dabei der jeweiligen Innervation von Nervenwurzeln, Nervenplexus bzw. peripheren Nerven. Da der erste Ast einer Nervenwurzel zur Rückenmuskulatur abzweigt, lässt sich ein segmentaler PSA-Befall der Rückenmuskulatur nutzen, um eine radikuläre Läsion zu sichern [30]. Dabei ist zu berücksichtigen, dass nur für die tiefer gelegenen Multifidi eine monosegmentale Innervation anzunehmen ist [31]. Auf ein EMG der Rückenmuskulatur sollte im Falle einer zurückliegenden Operation an der Wirbelsäule im betroffenen Gebiet verzichtet werden, da perioperative Nervenastläsionen zu falsch-positiven Befunden führen könnten. Ferner sollte auf die Absicherung des monoradikulären Befalls Wert gelegt werden, etwa indem einige Etagen oberhalb bzw. unterhalb des betroffenen Segmentes nach PSA gesucht wird.

Es gibt deutliche Belege dafür, dass die paravertebrale Muskulatur Radikulopathien elektromyographisch sensitiver sichern hilft als die Extremitätenmuskulatur [32]. Als Kritik an der Untersuchung paravertebraler Muskeln wird angeführt, dass PSA der paravertebralen Muskulatur bei Menschen mit einem Diabetes mellitus sowie bei älteren gesunden Personen unspezifisch gehäuft anzutreffen wäre [30]. Aus unserer Sicht trifft dies nicht zu. Wir gehen vielmehr davon aus, dass es sich um den relativ häufigen Fall asymptomatischer oder oligosymptomatischer Fälle von etwa einer PROMM oder anderen leichteren Fällen muskeldystrophischer Erkrankungen handelt, die mithilfe moderner genetischer Verfahren zunehmend besser diagnostisch erfasst und zugeordnet werden können.

Für Plexusneuropathien spielt neben dem PSA-Verteilungsmuster in der Extremitätenmuskulatur die Abwesenheit von PSA in paravertebraler Muskulatur eine wesentliche diagnostische Rolle [33]. Bei akuten entzündlichen Plexusneuropathien, wie etwa dem Parsonage-Turner-Syndrom oder der Lyme-Plexusneuritis, spielt PSA aus unserer Erfahrung aufgrund des verzögerten Auftretens gegenüber klinischen, bildgebenden und liquordiagnostischen Befunden oft eine untergeordnete Rolle. Allerdings gibt es leichtere oder atypische Fälle von Plexusneuropathien, in denen die akute Diagnostik nicht ergiebig ist. In diesen Fällen kann das PSA-Verteilungsmuster durchaus zur Diagnosefindung beitragen. Bei länger bestehender Symptomatik, wie etwa beim Thoracic-outlet-Syndrom oder der radiogenen Plexusneuropathie, spielt die PSA neben der Potenzialanalyse motorischer Einheiten eine wesentliche Rolle [34]. Im Fall des Arteria-spinalis-anterior-Syndroms kann PSA dabei helfen, die Ausdehnung der Rückenmarksläsion zu charakterisieren. Nämlich dann, wenn eine ischämische Rückenmarksläsion mittels Magnetresonanztomographie nicht fassbar ist. Gerade beim häufigen thorakalen bzw. thorakolumbalen Befallsmuster beim Arteria-spinalis-anterior-Syndrom trägt das Vorliegen von PSA zur Diagnosesicherung erheblich bei [35].

Bei einer PNP kann das Vorliegen von PSA für die Abschätzung der Akuität der Erkrankung herangezogen werden. Meist sind PNP relativ langsam progrediente Prozesse, bei denen PSA eher ungewöhnlich und wenn überhaupt, dann nur spärlich vorhanden ist. Es gibt jedoch auch akute oder subakute Formen der PNP (z. B. Guillain-Barré-Syndrom, chronische inflammatorische demyelinisierende Polyneuropathie (CIDP), multifokale motorische Neuropathie (MMN), Critical-illness-PNP, PNP bei rheumatischen Erkrankungen). Hier darf aus unserer Erfahrung mit mäßig oder gar stärker ausgeprägter PSA gerechnet werden. In unseren Händen findet sich bei sog. distal symmetrischen PNP unklarer Pathogenese nicht selten PSA in der Rückenmuskulatur, insbesondere am thorakolumbalen Übergang. Sofern ebendort Hinweise für einen neurogenen Umbau der PME fehlen, kann dieser Befund als möglicher Hinweis dafür dienen, dass es sich nicht um eine isolierte PNP handelt, sondern um eine multisystemische Erkrankung mit Beteiligung sowohl der peripheren Nerven als auch der Muskulatur. Dies kommt u. a. bei Kollagenosen vor sowie auch bei hereditären NME (z. B. proximale myotone Myopathie, M. Pompe). Darüber hinaus gehört PSA zur diagnostischen Absicherung der Vorderhornbeteiligung bei motorischen Systemerkrankungen (z. B. ALS). Die im Krankheitsverlauf ubiquitär nachweisbare PSA findet man neben dem ebenfalls ubiquitär fassbaren neurogenen Umbau mit persistierendem Sprouting-Phänomen bei kaum einer anderen Erkrankung als der ALS.

### Myogene Prozesse

Anders als bei motorisch-axonaler (neurogener) Degeneration sind Vorkommen und Befallsmuster von PSA bei primär myogenen Erkrankungen recht heterogen (Abb. 2). Zu den Myopathien, bei denen PSA kaum eine Rolle spielt, gehören u. a. die Steroidmyopathie, die Glykogenose Typ V (Mc Ardle), die kongenitalen Myopathien aufgrund von Mutationen im Ryanodin-Rezeptor‑1 sowie kongenitale Nemalin-Myopathien [36]. Hingegen wird bei inflammatorischen Myopathien in proximalen Muskeln PSA regelmäßig angetroffen, allen voran bei der Polymyositis sowie der Dermatomyositis [37]. Interessanterweise kann in den wenigen Fällen ausgeprägter Statin-Myopathien PSA ähnlich verteilt sein wie bei Polymyositis [38]. Bei der Einschlusskörpermyositis findet sich der PSA-Befall initial vor allem in den Hand- und Fingerbeugern, im Quadrizeps sowie den Fußhebern [18, 37]. Demgegenüber findet sich PSA bei der distalen Myopathie vom Typ Welander initial eher in den Hand- und Fingerstreckern bzw. den Zehen- und Fußhebern [23] und bei Patienten mit Muskeldystrophien vom Typ Miyoshi (Dysferlinopathien bzw. Anoctamin-5-Myopathien) eher in den Fußsenkern [39].

Prospektive Vergleichsstudien zum Vorliegen von PSA in definierten Muskeln unter definierten Untersuchungsbedingungen (Zahl der Nadelinsertionen etc.) bei einem Spektrum verschiedener Myopathien liegen bislang nicht vor. Für eine Stichprobe verschiedener Myopathien haben Hanisch und Mitarbeiter in einer retrospektiven Analyse gezeigt, dass PSA bei einigen Myopathien häufiger anzutreffen ist als bei anderen [18]. Dabei fanden sich Fi und PSW wie auch HERE bei folgenden Erkrankungen regelmäßig: sporadische Einschlusskörpermyositis, PROMM, M. Pompe, Matrin-3-Myopathie sowie zentronukleäre Myopathie. Weniger oft und vor allem auch in geringerer Ausprägung zeigte sich PSA bei verschiedenen dystrophischen Myopathien, wie etwa der fazioskapulohumeralen Dystrophie oder bei verschiedenen Gliedergürtelmuskeldystrophien [18].

Es gibt relativ wenige elektromyographische Untersuchungen, die sich der Frage der diagnostischen Wertigkeit unterschiedlicher Muskeln bei Patienten der gleichen Muskelerkrankung gewidmet haben. Hanisch und Mitarbeiter haben gefunden, dass PSA bei PROMM, M. Pompe, Matrin-3-Myopathie sowie zentronukleärer Myopathie besonders häufig und ausgeprägt in der Rückenmuskulätur zu detektieren war [18]. In einer weiteren Arbeit von Hanisch und Mitarbeitern wurde dies bereits für die myofibrillären Mopathien bestätigt, also Erkrankungen auf Grundlage von Mutationen in den Genen für Myotilin, Desmin, Filamin‑C und ZASP [19]. Eine umfangreiche Analyse zu PSA in verschiedenen Muskeln bei Patienten mit M. Pompe hat eindrücklich bestätigt, dass PSA in der Rückenmuskulatur deutlich häufiger zu finden ist als in der Extremitätenmuskulatur [40]. Auch in einer Arbeit zur Matrin-3-Myopathie wurde gezeigt, dass Fi, PSW und HERE, abgesehen von distaler Muskulatur in der paraspinalen Muskulatur besonders häufig nachweisbar sind [41]. Bei PROMM-Patienten, bei denen PSA nicht fassbar war, ist in der Regel die Rückenmuskulatur nicht mit EMG untersucht worden [6]. Diese Beispiele zeigen, dass der Rückenmuskulatur zur PSA-Detektion ein besonderer Stellenwert zukommt. Bei der Diagnostik myopathischer Erkrankungen erscheint die paravertebrale Muskultur am thorakolumbalen Übergang nicht nur hinsichtlich der Analyse motorischer Einheitenpotenziale die „vergessene Muskulatur“ zu sein, sondern gerade auch bei der sensitiveren PSA-Analyse. Nicht wenige Myopathiepatienten werden nicht durch Paresen klinisch auffällig, sondern durch Myalgien, Crampi oder Hyper-CK-Ämie. Wir möchten aufgrund der hier vorgelegten Befunde dazu anregen, bei diesen Patienten neben der Extremitätenmuskulatur regelmäßig auch die Rückenmuskulatur am thorakolumbalen Übergang in den elektromyographischen Untersuchungsgang mit einzubeziehen.

Da die EMG-Untersuchung der Rückenmuskulatur noch nicht allseits zur Routine gehört, möchten wir an dieser Stelle kurz skizzieren, wie wir es machen. Der Patient wird seitlich gelagert, und zwar in einer „Embryo“-Stellung, also mit maximaler Beugung in Hüfte und Knien und maximaler ventraler Beugung der gesamten Wirbelsäule. Der Kopf wird (falls möglich) maximal gebeugt auf eine weiche Rolle gelegt. Wichtig erscheint es, dass die Arme locker und im Ellenbogen gebeugt vor dem Bauch liegen. Keineswegs sollen die Hände am Knie gehalten werden, weil dabei oft eine Anspannung der Schulter- und Rückenmuskulatur ausgelöst wird. Wenn dabei keine hinreichende Entspannung der Rückenmuskulatur erzielt wird, hilft es in der Regel, wenn der Untersucher mit den Händen oberen Rücken und Knie des Patienten etwas gegeneinander drückt. Dabei wird die Rückenbeugung verstärkt und die Entspannung der Rückenmuskulatur gefördert. Sodann wird z. B. am thorakolumbalen Übergang der Raum zwischen zwei Dornfortsätzen getastet. Die Nadel wird etwa 2 Querfinger lateral dieses Raumes senkrecht eingestochen.

## Zusammenfassung

Die Art, das Verteilungsmuster sowie der zeitliche Verlauf muskulärer SA sind für die Diagnostik von NME bedeutsam, insbesondere bei der Detektion von NME im klinischen Alltag. Bei neurogenen Läsionen mit motorisch axonaler Beteiligung ist PSA meist 2 bis 4 Wochen nach Läsionsbeginn sicher fassbar. Das Verteilungsmuster korreliert dabei mit dem Läsionsort. Myopathien segregieren in solche mit einer hohen diagnostischen Wertigkeit von PSA und andere mit einer geringen Wertigkeit von PSA. Ein Schwerpunkt der vorliegenden Arbeit lag dabei in der Darstellung der unterschiedlichen PSA-Verteilungsmuster bei myogenen Erkrankungen.
